# B procyanidins of *Annona crassiflora* fruit peel inhibited glycation, lipid peroxidation and protein-bound carbonyls, with protective effects on glycated catalase

**DOI:** 10.1038/s41598-019-55779-3

**Published:** 2019-12-16

**Authors:** Allisson B. Justino, Rodrigo R. Franco, Heitor C. G. Silva, André L. Saraiva, Raquel M. F. Sousa, Foued S. Espindola

**Affiliations:** 1 Institute of Biotechnology, Federal University of Uberlandia, Av. Pará, 1720, 38400-902, Uberlandia/MG, Brazil; 2 Institute of Chemistry, Federal University of Uberlandia, Av. João Naves de Ávila, 2121, 38408-100 Uberlândia/MG, Brazil

**Keywords:** Biochemistry, Biological techniques, Plant sciences

## Abstract

Advanced glycation end-products (AGEs) have been reported as results of increased oxidative stress. Consequently, the search for new antioxidant and anti-glycating agents is under intense investigation. Plant-derived procyanidins have previously demonstrated anti-glycation properties. Thus, this study aimed to isolate procyanidins from *Annona crassiflora* fruit peel, a species from the Brazilian Savanna, and investigate their antioxidant and anti-glycation effects. Free radical scavenging and quenching properties, formation of reactive oxygen species (ROS), AGEs, protein carbonyl and thiol groups, lipid peroxidation, crosslinked AGEs, as well as glycated catalase activity, were analyzed. In addition, *in silico* assessment of absorption, distribution, metabolism, excretion and toxicity was carried out. The procyanidins-enriched fraction, named here as F7, showed high antioxidant and anti-glycation capacities, with inhibitory activities against lipid peroxidation, and AGEs and ROS formation. In addition, there were reductions in AGEs-induced crosslinks and protein carbonyls and protective effects against oxidation of thiol groups and glycated-catalase. ADMET predictions of F7 showed favorable absorption and distribution, with no hepatotoxicity or mutagenicity. Together, our results support the anti-glycation activities of the procyanidins-enriched fraction from *A. crassiflora*, and suggest that these effects are triggered, at least in part, by scavenging free radical and dicarbonyls intermediates.

## Introduction

Glycation is a non-enzymatic reaction involving reactive carbonyls of sugars with amino groups of a protein, lipid or nucleic acid generating Schiff bases, which rearrange to Amadori products^1^. Due to instability of Amadori products, further rearrangement reactions occur that lead to the formation of advanced glycation end-products (AGEs)^2^. The damaging potential of AGEs results from direct alterations on protein structures and functions due to AGEs per se or the cross-linking effect of some AGEs. Recently, AGEs have been reported as results of increased oxidative stress, since many of them are generated by a combination of oxidation and glycation processes^3–5^. Oxidative stress is a result of an imbalance between free radical production and antioxidant defense mechanisms^6^. Moreover, it is well known that AGEs are increasingly formed under hyperglycemic conditions^2^. Thus, oxidative stress, reactive oxygen species (ROS) and AGEs are involved in the pathogenesis of diabetes *mellitus* and its complications^7,8^.

Natural products are considered important sources of investigation of potential new drugs, and studies of bioactive molecules effective in the control of oxidative stress and non-enzymatic glycation are promising. Plants with known antioxidant and anti-glycation properties are receiving a lot of attention as they are believed to have less adverse effects^9^. Proanthocyanidins, a class of flavonoids, are the most studied secondary metabolites found in plants due to their antioxidant properties^10–12^. Recent studies have shown the anti-glycation potential of procyanidins, oligomeric natural compounds belonging to the class of proanthocyanidins formed from catechin and epicatechin molecules^13,14^.

The fruit peel of *Annona crassiflora* Mart., a native species of the Brazilian Savanna biome belonging to the Annonaceae family, has been recently shown by our group as a source of proanthocyanidins^15^. Different parts of this species are popularly used for treating wounds, rheumatism, venereal diseases, diarrhea, snakebites and microbial infections^16,17^. Previously, we conducted a pre-purification of the crude ethanol extract of *A. crassiflora* fruit peel, obtaining non-cytotoxic polyphenols fractions (EtOAc and n-BuOH)^15^. Additionally, we related the inhibition of glycation by EtOAc fraction. However, only the preliminary BSA-fructose model was tested without focusing on specific aspect of AGE inhibition and its mechanism.

Thus, the present study aimed to purify procyanidins from *A. crassiflora* fruit peel with antioxidant and anti-glycation effects by examining free radical scavenging and quenching properties, ROS, AGEs, protein carbonyl and thiol groups formation, lipid peroxidation, crosslinked AGEs, as well as protective effect on glycated catalase. In addition, *in silico* assessment of the absorption, distribution, metabolism, excretion and toxicity (ADMET) profile of the main procyanidins was carried out.

## Results

### Extraction yield

The ethanolic extraction of *A. crassiflora* fruit peel had a total yield of about 5%. Among the organic fractions from ethanol extract, EtOAc fraction presented a yield of approximately 5.5%. Proanthocyanidins were purified from EtOAc fraction by column chromatography, which resulted in the grouping of 12 fractions (F1-F12) according to the R_f_ values. F6 and F7 showed higher yields, 24.6 and 14.0%, respectively, followed by F4, F5, F8 and F3 (5.2, 3.7, 1.6 and 1.4%, respectively). The other fractions had yields lower than 1%.

### Phytochemical prospection

Total phenolics and proanthocyanidins contents of the purified proanthocyanidins fractions from *A. crassiflora* fruit peel are shown in Table 1. In general, all fractions presented amount of total phenolics and proanthocyanidins higher than 150 mg GAE g^−1^ and 250 mg CE g^−1^, respectively. As expected, EtOAc fraction also contained a substantial amount of phenolic compounds. Among the proanthocyanidins fractions, F7 had the highest values of total phenolics (660.6 mg GAE g^−1^). In addition, proanthocyanidins were concentrated mainly in F7 fraction (1295.7 mg CE g^−1^).

**Table 1:** Total phenols and proanthocyanidins contents of fractions from *A. crassiflora* fruit peel. Values expressed as mean ± standard deviation. Note: EtOAc: ethyl acetate fraction.

Fractions	Total phenolic content (mg GAE g^−1^)	Proanthocyanidins content (mg CE g^−1^)
F1	205.5 ± 15.1	385.3 ± 19.5
F2	483.9 ± 52.7	720.4 ± 66.8
F3	446.9 ± 33.4	650.2 ± 54.2
F4	468.9 ± 24.2	840.2 ± 95.3
F5	502.1 ± 16.4	902.7 ± 126.5
F6	545.1 ± 65.8	1102.2 ± 135.7
F7	660.6 ± 71.9	1295.7 ± 91.4
F8	455.8 ± 32.5	878.3 ± 165.8
F9	448.8 ± 22.3	778.4 ± 116.0
F10	384.1 ± 53.6	628.8 ± 49.0
F11	232.8 ± 32.4	443.0 ± 31.8
F12	151.3 ± 14.0	278.3 ± 31.8
EtOAc	497.9 ± 38.7	757.5 ± 12.0

Because F7 had the highest value of proanthocyanidins content, this fraction was subjected to a HPLC-ESI-MS/MS analysis in order to identify its main proanthocyanidins. According to the high-resolution spectra of the molecular ions, error values (ppm), retention times, sequential mass spectra and comparisons with data from the literature^15,18^ and Metlin library, we identified oligomers of B-type procyanidin, including dimer, trimer, tetramer and pentamer (Table 2). The chromatogram and sequential mass spectra can be found as Supplementary material (Figs. S1–S6).

**Table 2:** Proanthocyanidins identified in the proanthocyanidins-enriched fraction from *A. crassiflora* fruit peel by HPLC-ESI-MS/MS (negative mode).

Compound identified	Retention time (min)	Formula	Mass calculated	*m/z*	Error (ppm)	*m/z* of fragments	References
B-type procyanidin (dimer)	2.6–5.0	C_30_H_25_O_12_^−^ [M-H]^−^	577.1351 [M-H]^−^	577.1354 [M-H]^−^	0.51	451, 407, 339, 289, 245, 243, 205, 203, 161, 125	^15,18^
B-type procyanidin (trimer)	3.0–4.8	C_45_H_37_O_18_^−^ [M-H]^−^	865.1985 [M-H]^−^	865.1984 [M-H]^−^	−0.11	695, 577, 425, 363, 289, 287, 204, 203, 125	^18^
B-type procyanidin (tetramer)	3.0–4.7	C_60_H_49_O_24_^−^ [M-H]^−^C_30_H_24_O_12_^−^ [M-2H]^2−^	1153.2619 [M-H]^−^576.1279 [M-2H]^2−^	1153.2614 [M-H]^−^576.1275 [M-2H]^2−^	−0.43–0.34	863, 739, 577, 575, 545, 449, 407, 339, 289, 245, 243, 205, 203, 125	^18^
B-type procyanidin (pentamer)	3.7–4.7	C_75_H_61_O_30_^−^ [M-H]^−^C_37.5_H_30_O_15_^−^ [M-2H]^2−^	1441.3253 [M-H]^−^720.1590 [M-2H]^2−^	1441.3255 [M-H]^−^720.1573 [M-2H]^2−^	0.13–0.27	720, 693, 577, 575, 451, 407, 289, 217, 125	^18^

### Antioxidant capacity

**Figure 1:**
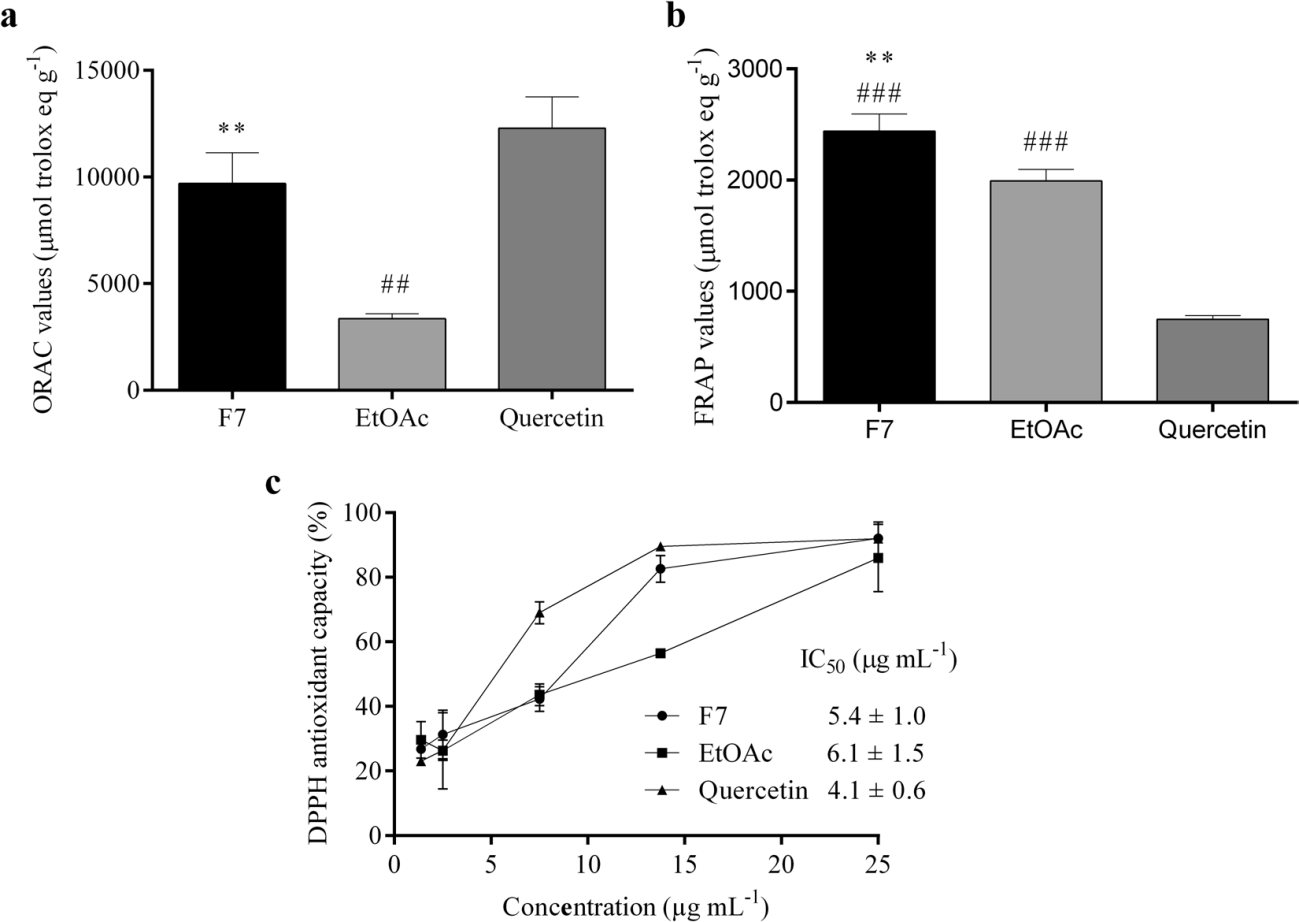
Antioxidant capacity analysis of proanthocyanidins-enriched and EtOAc fractions from *A. crassiflora* fruit peel and quercetin by ORAC (**a**), FRAP (**b**) and DPPH (c) methods. Values (mean ± standard deviation) are expressed as µmol trolox equivalents g^−1^ (**a**,**b**), percentage of antioxidant activity and IC_50_ values. (**c**) Note: F7: procyanidins-enriched fraction, EtOAc: ethyl acetate fraction. ^**^p < 0.01 versus EtOAc; ^##^*p* < 0.01 and ^###^*p* < 0.001 versus quercetin (control).

In all antioxidant capacity assays, F7 had the highest values (data not shown). Because of this, the biological assays were performed with F7 and EtOAc fractions, and quercetin as control. In the ORAC assay, F7 had higher antioxidant capacity than EtOAc (9691 µmol trolox eq g^−1^, *p* < 0.01), with a similar value to quercetin (12283 µmol trolox eq g^−1^) (Fig. 1a). In relation to the FRAP method, F7 showed higher antioxidant activity than EtOAc and quercetin (2438 µmol trolox eq g^−1^, *p* < 0.01) (Fig. 1b). In addition, the analysis using DPPH antiradical test showed that F7 and EtOAc had a high antioxidant capacity (IC_50_ 5.4 and 6.1 µg mL^−1^, respectively). It is worth emphasizing that these fractions showed similar results to quercetin in the DPPH method (IC_50_ 4.1 µg mL^−1^) (Fig. 1c).

**Figure 2:**
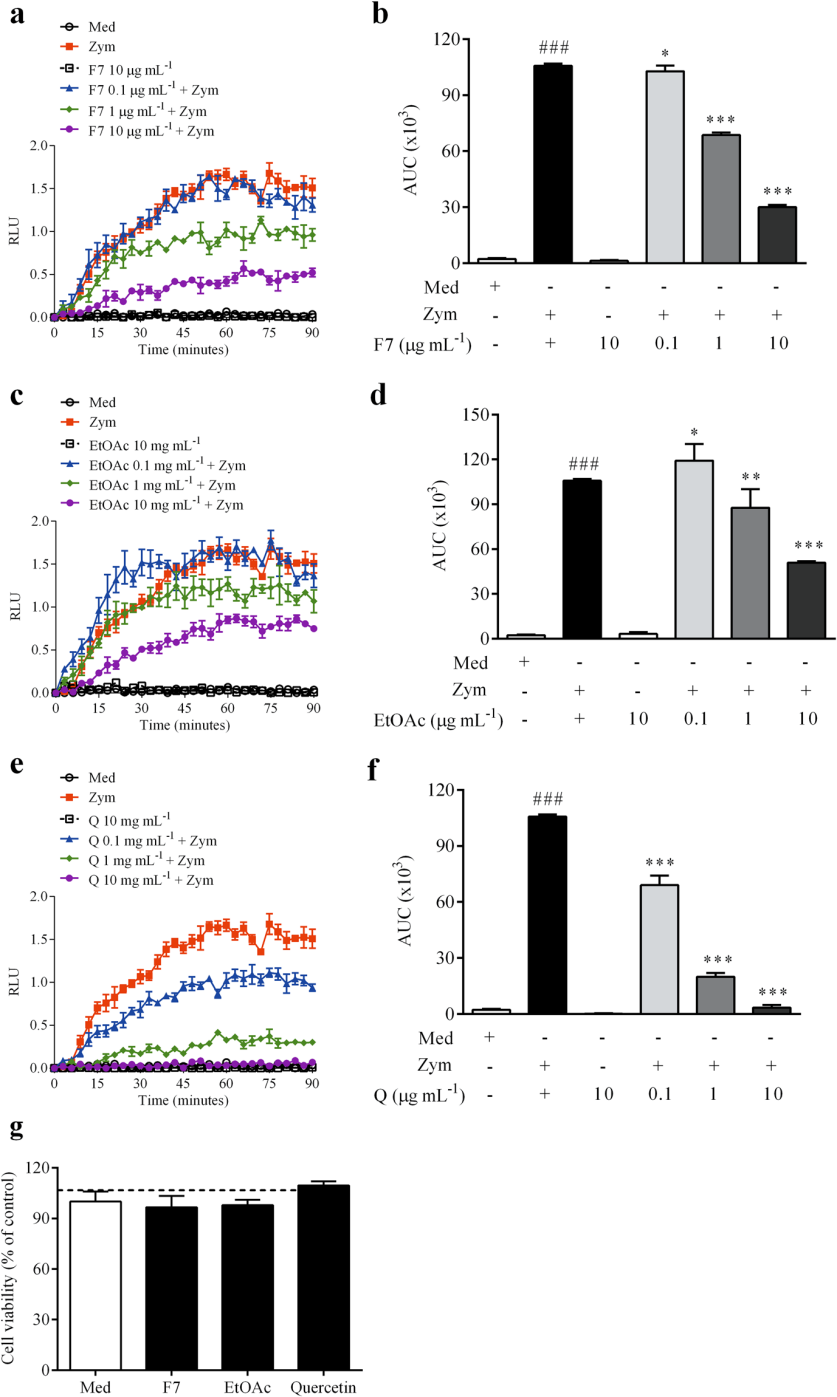
ROS production and cell viability analyzes using the proanthocyanidins-enriched and ethyl acetate fractions from *A. crassiflora* fruit peel and quercetin in macrophages. Values (mean ± standard deviation) are expressed as relative luminescent units (RLU) for F7 (**a**), EtOAc (**c**) and quercetin (**e**), and area under the curve (AUC) for F7 (**b**), EtOAc (**d**) and quercetin (**f**). (**g**) Shows cellular viability of macrophages treated with F7, EtOAc and quercetin (10 µg mL^−1^). Note: F7: proanthocyanidins-enriched fraction, EtOAc: ethyl acetate fraction, Q: quercetin, Med: medium, Zym: zymosan. ^*^p < 0.05, ^**^p < 0.01 and ^***^p < 0.001 versus Zym; ^###^*p* < 0.001 versus Med.

The cell viability and reduced of ROS production using the F7 and EtOAc fractions are shown in Fig. 2. As expected, F7, EtOAc and quercetin did not stimulate ROS production. F7 was able to reduce ROS production at the concentrations of 0.1, 1 ad 10 µg mL^−1^ (*p* < 0.05) (Fig. 2a,b), whereas that EtOAc reduced ROS production only at 1 and 10 µg mL^−1^ (*p* < 0.05) (Fig. 2c,d). Quercetin also reduced ROS production at all concentrations tested (*p* < 0.001) (Fig. 2e,f). The results also showed that F7 and EtOAc fractions were not cytotoxic in the concentration of 10 μg mL^−1^ (Fig. 2g).

### Inhibition of lipid peroxidation

**Figure 3:**
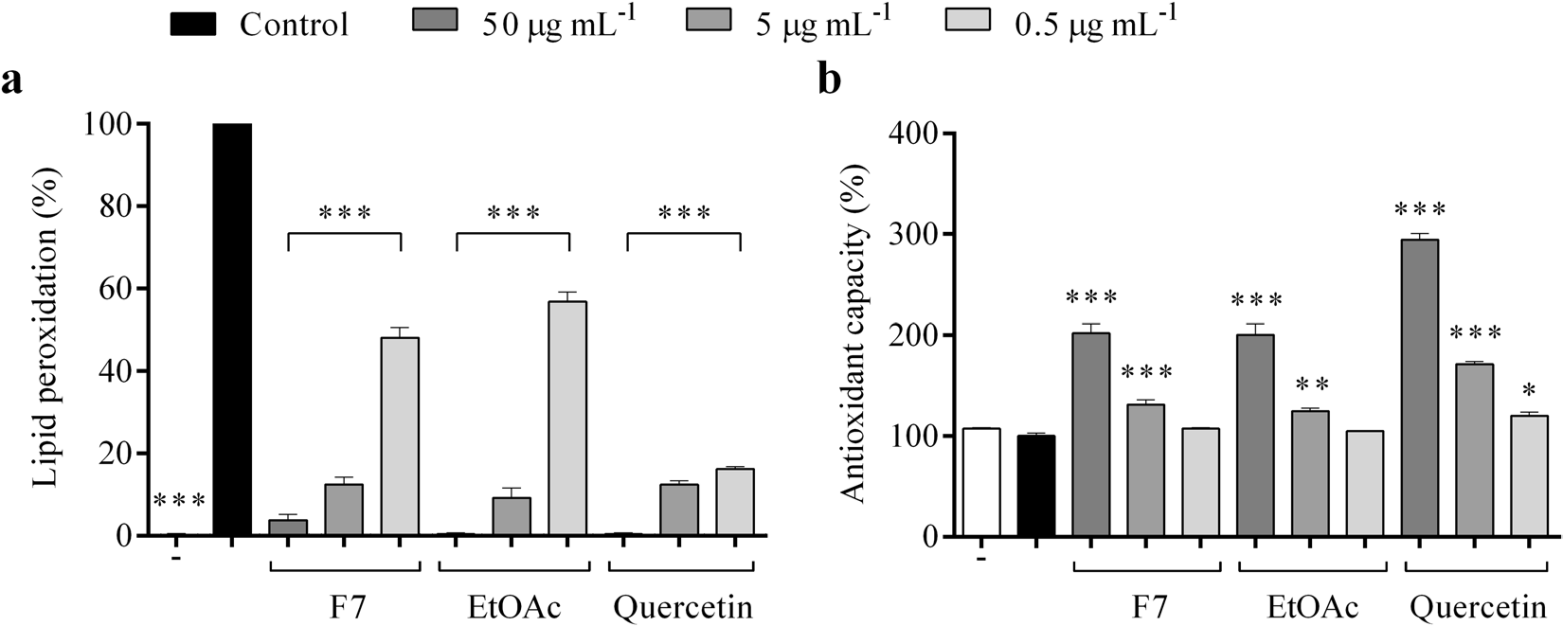
Analysis of lipid peroxidation (**a**) and total antioxidant capacity (**b**) in the livers of healthy rats treated for 60 minutes with 0.5, 5 and 50 µg mL^−1^ of proanthocyanidins-enriched and EtOAc fractions from *A. crassiflora* fruit peel and quercetin. Values expressed as mean ± standard deviation. Note: F7: proanthocyanidins-enriched fraction, EtOAc: ethyl acetate fraction. ^*^p < 0.05, ^**^p < 0.01 and ^***^p < 0.001 versus control.

The addition of F7, EtOAc and quercetin to the liver lipid peroxide induced by FeSO_4_-ascorbate significantly reduced TBARS formation. All concentrations of F7, EtOAc and quercetin (0.5, 5 and 50 µg mL^−1^) reduced lipid peroxidation (*p* < 0.001) (Fig. 3a). Furthermore, F7 and EtOAc at 5 and 50 µg mL^−1^ increased total antioxidant capacity in the liver (*p* < 0.01) (Fig. 3b).

### Inhibition of the formation of advanced glycation end products (AGEs)

**Figure 4:**
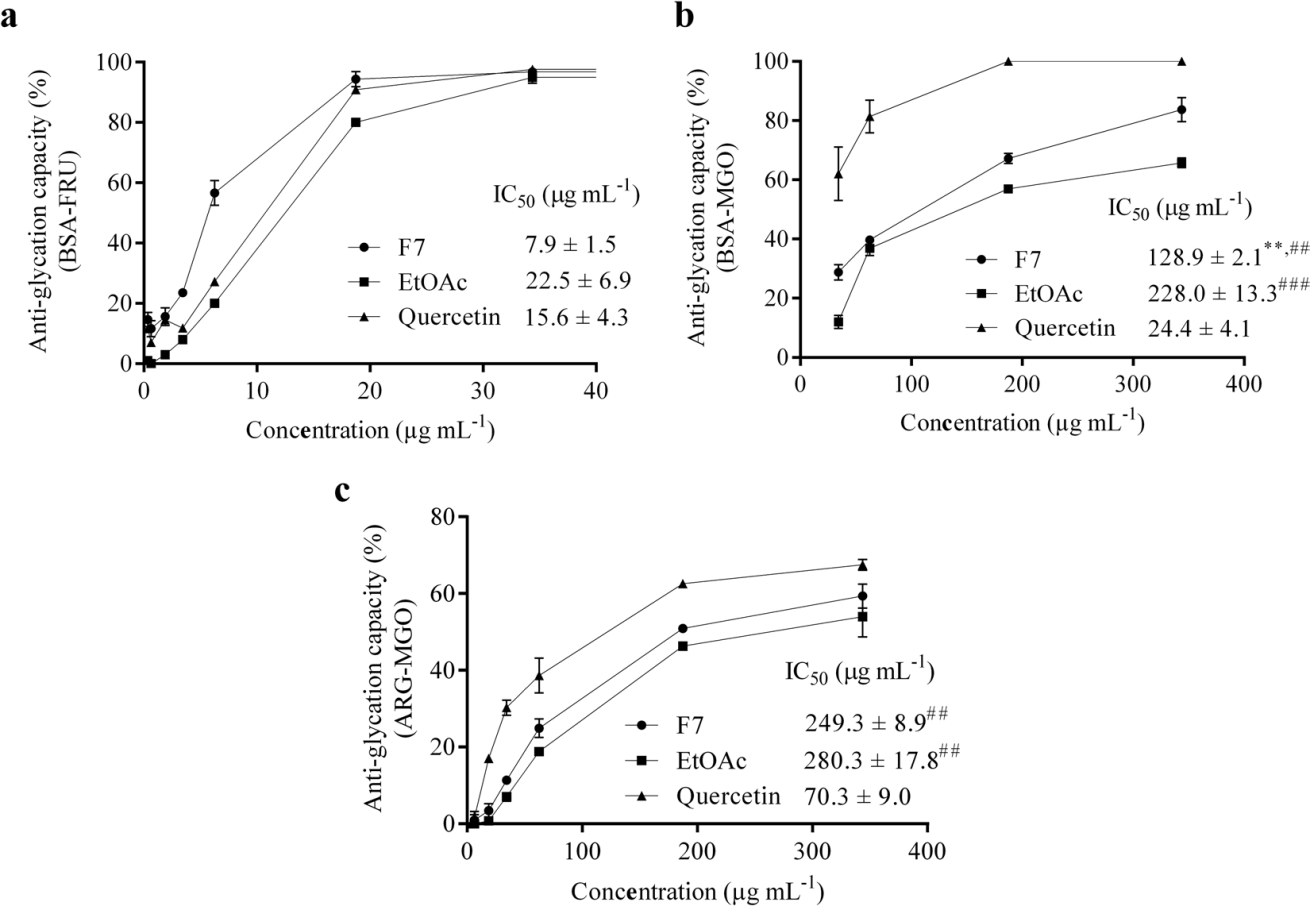
Glycation inhibitory activity analysis of the proanthocyanidins-enriched and EtOAc fractions from *A. crassiflora* fruit peel and quercetin in BSA-fructose (BSA-FRU) (**a**), BSA-methylglyoxal (BSA-MGO) (**b**) and arginine-methylglyoxal (ARG-MGO) (**c**) models. Values (mean ± standard deviation) are expressed as a percentage of glycation inhibition and IC_50_ values. Note: F7: proanthocyanidins-enriched fraction, EtOAc: ethyl acetate fraction. ^**^p < 0.01 versus EtOAc; ^##^*p* < 0.01 and ^###^*p* < 0.001 versus quercetin (control).

In the BSA-fructose model, F7 was able to inhibit glycation over 90% at 20 μg mL^−1^, as well as quercetin; EtOAc at 20 µg mL^−1^ reduced glycation over 80% (Fig. 4a). It is worth mentioning that their IC_50_ values did not present significant differences. In addition, F7 inhibited fluorescent AGE production by over 80% at 345 µg mL^−1^ in BSA-methylglyoxal assay, with an IC_50_ value lower than EtOAc (128.9 µg mL^−1^, *p* < 0.01) (Fig. 4b). Anti-glycation assay in arginine–methylglyoxal model showed that F7 and EtOAc inhibited fluorescent AGE generation over 50% at 345 µg mL^−1^, presenting no difference between their IC_50_ values (249.3 and 280.3 µg mL^−1^, respectively) (Fig. 4c). Quercetin showed the lowest IC50 values in BSA-methylglyoxal and arginine-methylglyoxal assays (24.4 µg mL^−1^). The anti-glycation results obtained for F7 and EtOAc fractions showed significant high correlations between BSA-fructose and BSA-methylglyoxal (*r* = 0.66, *p* < 0.05), BSA-fructose and arginine-methylglyoxal (*r* = 0.75, *p* < 0.05) and BSA-methylglyoxal and arginine-methylglyoxal (*r* = 0.96, *p* < 0.05) methods. A high correlation was also observed for F7 and EtOAc between non-enzymatic glycation and lipid peroxidation assays (*r ~* 0.72, *p* < 0.05).

### Glycation-induced protein carbonyl and thiol group formation

**Figure 5:**
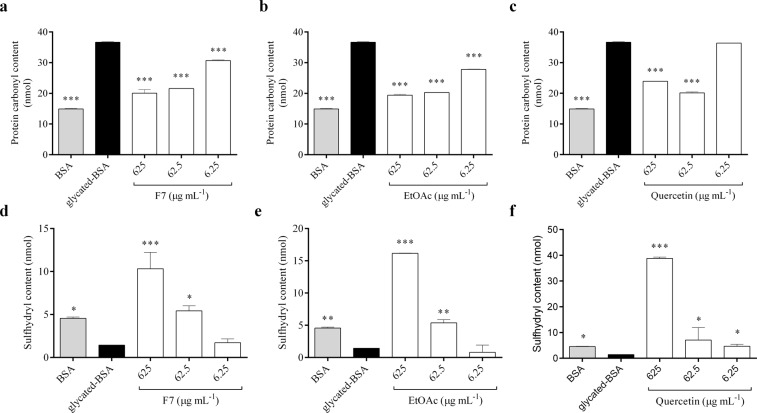
Effects of proanthocyanidins-enriched and EtOAc fractions from *A. crassiflora* fruit peel and quercetin on protein carbonyl (**a**–**c**, respectively) and thiol (**d**–**f**, respectively) groups of fructose-modified BSA. Data are presented as mean ± standard deviation. Note: F7: proanthocyanidins-enriched fraction, EtOAc: ethyl acetate fraction. ^*^p < 0.05, ^**^p < 0.01 and ^***^p < 0.001 versus glycated-BSA (control).

BSA glycated for 4 days showed an increase in protein carbonyl groups compared to BSA alone (*p* < 0.001) (Fig. 5). Compared to glycated BSA, both F7 and EtOAc inhibited formation of protein-bound carbonyls groups at all concentrations tested (6.25, 62,5 and 625 µg mL^−1^, *p* < 0.001) (Fig. 5a,b). Quercetin reduced carbonyls groups in BSA only at 62.5 and 625 µg mL^−1^) (Fig. 5c). Glycated BSA had a lower thiol content compared to BSA alone (*p* < 0.05) (Fig. 5). F7 and EtOAc had a protective effect against oxidation of thiol groups (*p* < 0.05), as well as quercetin, when compared to glycated BSA (Fig. 5d–f). Also, the anti-glycation properties of F7 and EtOAc fractions were correlated with their capacity to inhibit protein carbonylation (*r* ~ 0.75, *p* < 0.05).

### Crosslinked advanced glycation end products (AGEs)

**Figure 6:**
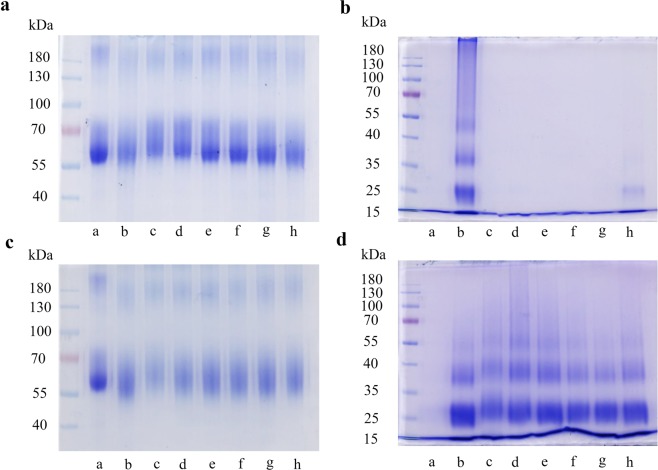
SDS-PAGE gels showing the effects of the proanthocyanidins-enriched fraction from *A. crassiflora* fruit peel (F7) on formation of crosslinked AGEs formed by glycation of BSA (10 mg mL^−1^) and lysozyme (10 mg mL^−1^) by fructose (**a**,**b**, respectively) or methylglyoxal (**c**,**d**, respectively) for 4 weeks at 37 °C. Unmodified BSA and lysozyme (lane a), glycated BSA and lysozyme (lane b) or BSA and lysozyme glycated in the presence of 625 (lane c), 345 (lane d), 187.5 (lane e), 62.5 (lane f), 34.5 (lane g) and 18.75 µg mL^−1^ (lane h) of F7.

SDS-PAGE gels showing the effects of F7 on formation of crosslinked AGEs formed by glycation of BSA and lysozyme by fructose or methylglyoxal are shown in Fig. 6. A major band around 66 kDa with high density was observed for native BSA (Fig. 6a,c, lane a), but nevertheless this band density was low in glycated BSA (Fig. 6a,c, lane b). F7 was able to slightly increase density of the 66 kDa band of BSA glycated by fructose (Fig. 1, lanes c–h). However, F7 seems to have no effect on BSA glycated by methylglyoxal (Fig. 1c). Furthermore, glycation of lysozyme by fructose or methylglyoxal produces crosslinked AGEs that present as dimers, with approximate molecular weights of 28 and 36 kDa, as shown in Fig. 6b,d (lane b), when compared to native lysozyme (Fig. 6b,d, lane a). F7 reduced AGE-induced dimerization of lysozyme in a dose-dependent manner (Fig. 6b,d, lanes c–h). These results were similar to the quercetin (Supplementary Material, Fig. S7).

### Glycated catalase activity and fluorescence

**Figure 7:**
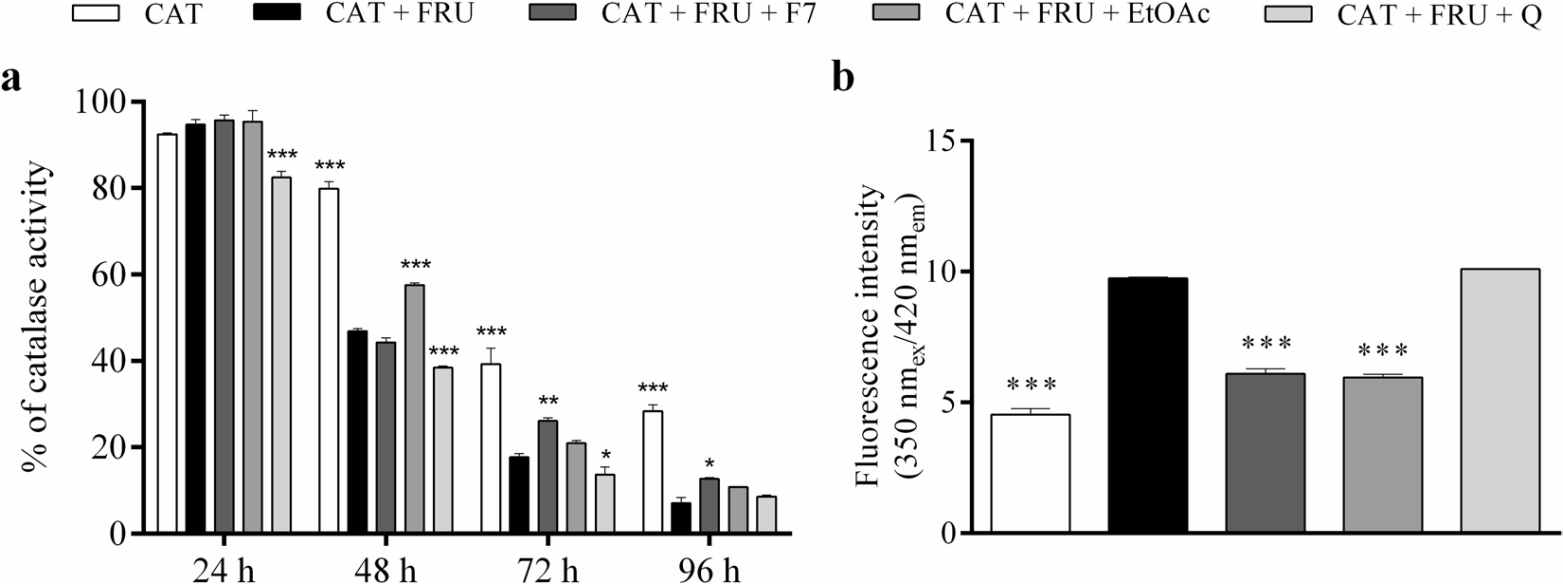
Activity (**a**) and fluorescence intensity (**b**) of catalase in the presence of 16 mmol L^−1^ fructose and 3 µg mL^−1^ proanthocyanidins-enriched and EtOAc fractions from *A. crassiflora* fruit peel and quercetin, for 96 h. H_2_O_2_ was used as substrate for catalase activity assay. Note: CAT: catalase, FRU: fructose, F7: proanthocyanidins-enriched fraction, EtOAc: ethyl acetate fraction and Q: quercetin. ^*^p < 0.05, ^**^p < 0.01 and ^***^p < 0.001 versus CAT + FRU.

The effect of F7 and EtOAc on activity and fluorescence of glycated catalase is shown in Fig. 7. Effect of 16 mmol L^−1^ fructose as glycating agent on the catalytic activity of catalase was evaluated for 96 h (Fig. 7a). As expected, high concentration of fructose caused a decrease in catalase activity compared with the control, representing catalytic activity of catalase without fructose before incubation (day 0). Catalytic activity of glycated catalase decreased to approximately 46% on the 2nd day, 16% on the 3rd day, and about 6% on the 4th day (Fig. 7a). This decrease in catalase activity was attenuated by EtOAc on the 2nd day (57%, *p* < 0.001), and by F7 on the 3rd and 4th days (26 and 13%, respectively, *p* < 0.05) (Fig. 7a). These results are in agreement with the fluorescent intensity values observed for glycated catalase and glycated catalase incubated with F7 and EtOAc for 96 h (Fig. 7b). An increase of fluorescence intensity (approximately 115%, *p* < 0.001) was observed for glycated catalase when compared with catalase alone. When catalase is incubated for 96 h at 37 °C with fructose and F7 or EtOAc, a decrease in AGE-specific fluorescence was observed (*p* < 0.001) (Fig. 7b). Quercetin was not able to increase the activity and reduce the fluorescent intensity of glycated catalase. To ensure F7 and EtOAc fractions do not have any effect on reducing hydrogen peroxide absorbance, hydrogen peroxide was placed in the presence of these fractions and no reduction in the absorbance value was observed.

### *In Silico* pharmacokinetic properties prediction

The prediction of pharmacokinetic properties of procyanidins of F7 is shown in Table 3. All compounds showed intestinal absorption above 40%, with the exception of B procyanidin pentamer (33%), and had no interference of metabolism by binding families of cytochrome P enzymes. The predicted intestinal absorption was based on the original compounds. The steady-state volume of distribution (VDss) values of F7 procyanidins range from −0.046 to 0.011, indicating uniform distribution. With regard to the total clearance, the larger oligomers showed the lowest values. Also, all procyanidins presented no hepatotoxicity or mutagenicity.

**Table 3:** *In silico* pharmacokinetics proprieties for B-type procyanidins (dimer, trimer, tetramer and pentamer)a.

Compound	Intestinal absorption (% absorbed)	VDss (log L kg^−1^)	CYP Inhibitor	Total clearance (log mL min^−1^ kg^−1^)	Hepatotoxicity	AMES toxicity
B-type procyanidin (dimer)	62.436	−0.046	No	0.192	No	No
B-type procyanidin (trimer)	52.759	0	No	−2.932	No	No
B-type procyanidin (tetramer)	43.41	0.011	No	−5.074	No	No
B-type procyanidin (pentamer)	33.737	0.011	No	−7.278	No	No

^a^Intestinal absorption, steady state volume of distribution (VDss), cytochrome P enzymes (CYP) inhibition, total clearance, hepatotoxicity and mutagenicity (AMES) analysis. Cytochrome P enzymes: CYP1A2, CYP2C19, CYP2C9, CYP2D6 and CYP3A4.

## Discussion

In this study, we performed an ethanolic extraction of *A. crassiflora* fruit peel, a liquid-liquid partitioning of the ethanol extract and a column chromatography of EtOAc in order to obtain purified proanthocyanidins. Previously, we identified various phenolic compounds in EtOAc, such as caffeoyl-glucoside, (epi)catechin, feruloyl-galactoside, quercetin-glucoside, kaempferol and procyanidin B2^15^. The column chromatography concentrated the proanthocyanidins, with F7 having the highest values of total phenolics and proanthocyanidins (Table 1). These results are in accordance with HPLC-ESI-MS/MS analysis, which indicated the presence of B-type procyanidins oligomers in F7, such as dimer, trimer, tetramer and pentamer (Table 2).

The high content of proanthocyanidins in F7 might explain its high antioxidant capacity. F7 had higher antioxidant capacities by the oxygen radical absorption capacity (ORAC), 2,2-diphenyl-1-picrylhydrazyl (DPPH) free radical scavenging and ferric reducing antioxidant power (FRAP) methods (Fig. 1). These methods used together reinforce its antioxidant properties, since they are based on different antioxidative reaction mechanisms involving hydrogen atom and electron transfer^19,20^. FRAP method evaluates the ability to transfer electrons, ORAC analyzes the hydrogen transfer potential, while DPPH assay detects both hydrogen and electrons transfer capacities^20,21^. In the FRAP assay, F7 and EtOAc had higher antioxidant activity than quercetin, which may indicate that these fractions have greater ability to reduce oxidants by electrons transfer than quercetin. However, in the ORAC assay, both quercetin and F7 showed similar antioxidant activity, suggesting similar hydrogen transfer capacity between them. The antiradical activity of proanthocyanidins is principally based on the redox properties of their hydroxyl groups^22^. Through the availability of their phenolic hydrogens, proanthocyanidins act as hydrogen donating radical scavengers and singlet oxygen quenchers and electron-donating agents, with properties to form stable antioxidant-derived radicals^11,23,24^. In addition, F7 was able to reduce zymosan-induced ROS production in macrophages with no cytotoxicity (Fig. 2). It is worth mentioning that cellular-based assays provide a more realistic model of the *in vivo* antioxidant capacity of phytochemicals.

It is well known that oxidative stress and increased free radical production induce lipid peroxidation, since lipids are most susceptible to the attack of reactive oxygen and nitrogen species^25^. This may compromise the functions of lipoproteins due to the decrease in membrane fluidity^25^. Also, lipid peroxidation leads to complex products, such as hydroperoxides, aldehydes and polymeric materials, which may exert cytotoxic and genotoxic effects^26^. The antioxidant potential of F7 contributed to the reduction of lipid peroxidation in the liver by Fe^2+^-ascorbate-induced lipid peroxidation assay (Fig. 3). This can be explained by the fact that F7 was able to control reactive oxygen species production. Previous studies have also demonstrated the potential of proanthocyanidins in reducing lipid peroxidation in diabetic animals^27,28^.

In addition to overproduction of ROS, increased AGEs formation are also usually manifested in oxidative stress conditions^4^. Free radicals could mediate the conversion of Amadori products to AGEs^29^. Previously, we demonstrated the antioxidant and anti-glycation potential of ethanol extract of *A. crassiflora* fruit peel and its EtOAc fraction^15^. However, we tested only on the BSA-fructose model. Here, we focused on some specific aspect of AGEs inhibition and its mechanism. Interestingly, F7 exhibited an inhibitory potential against AGEs formation in the BSA-fructose, BSA-methylglyoxal and arginine-methylglyoxal models, with IC_50_ values lower than EtOAc (Fig. 4). It is noteworthy that these models together evaluate all stages of protein glycation^30^. According to Wang, *et al*.^30^, the arginine model evaluates a major and specific source of AGE generate by reactive carbonyls, such as methylglyoxal, that in reaction with the guanidine group of arginine forms a fluorescent AGE called argpyrimidine. The argpyrimidine can accumulate in various tissues causing systemic inflammatory processes and cataracts in lens^31^. Thus, F7 may act on the middle and all stages of protein glycation, as well as on a major and specific source of AGEs production.

Fructose can be generated from sorbitol oxidation and its accumulation on ocular lens and peripheral nerves is due to a high blood glucose concentration^32^. Methylglyoxal is primarily generated as an intermediate in the glycolysis pathway, being an reactive carbonyl^33^. Both fructose and oxidation products such as methylglyoxal may promote glycation of proteins and lipids, with the consequent generation of AGEs and ROS^30^, which can lead to diabetic retinopathy and peripheral neuropathy^34,35^. Therefore, inhibition of non-enzymatic glycation mediated by fructose and its oxidant products is a viable strategy to prevent AGEs formation and the complications related to diabetes.

Protein oxidation has a key role in AGEs formation and together with glycation can form protein-bound carbonyl groups^36^. In this study, carbonyl groups were generated by incubating BSA with a high concentration of fructose. F7, as well as EtOAc, inhibited protein-bound carbonyl groups with the results comparable to the quercetin (Fig. 5). In addition, the glycation mediated accumulation of carbonyl groups in proteins may lead to protein cross-linking^14,37^, which was observed for BSA and lysozyme treated with fructose and methylglyoxal (Fig. 6). F7 was able to reduce AGE-induced dimerization of lysozyme in a dose-dependent manner, especially when incubated with fructose. Our results corroborate a study conducted by Muthenna, *et al*.^14^, which showed that a procyanidin-B2 fraction of cinnamon was able to inhibit glycation and protein cross-links, and to reduce carbonyl content of eyes lens. Furthermore, the loss of protein thiol groups also occurs in glycation, which may potentially lead to severe structural changes in various biological structures^38^. The oxidation of thiol groups was observed following glycation of BSA and this was inhibited by F7 and EtOAc fractions (Fig. 5). The carbonyl and thiol assays provide mechanistic information concerning the effects of the procyanidin-enriched fraction of *A. crassiflora* on AGEs.

In general, many antioxidant compounds, especially proanthocyanidins, show anti-glycation properties^39,40^, which are associated with the presence of hydroxyl groups^41^. Oligomeric procyanidins present higher antioxidant and glycation inhibitory activities than monomers^42,43^. They can reduce AGEs formation by different mechanisms, such as inhibiting ROS formation during non-enzymatic glycation, Schiff base and Amadori products, as well as blocking AGE-RAGE receptors, capturing precursors such as 1,2-dicarbonyls and/or interacting with glucose and preventing it from binding to proteins^14,44–46^. Our results suggest that the proanthocyanidins-enriched fraction might exert its anti-glycation activity by scavenging free radical and dicarbonyls intermediates.

Antioxidant enzymes have important roles in the defense system for controlling ROS production and repairing damage resulting from oxidative stress^47^. Catalase is one of the main enzymes involved in the elimination of ROS, particularly hydrogen peroxide, being highly glycated^48,49^. Glycation of catalase can lead to a decrease in its activity and, consequently, to an imbalance in oxidants and antioxidants homeostasis^50^. A decrease in catalase activity was observed in the presence of fructose. Our results showed that the catalase samples which contained F7 in addition to fructose had more enzyme activity compared with the same samples without the proanthocyanidins-enriched fraction after 72 and 96 h of incubation; this was observed for EtOAc at 48 h of incubation (Fig. 7). In addition, an increase of fluorescence intensity was observed when catalase was incubated with fructose for 96 h, indicating the presence of fluorescent compounds being formed during glycation^49,51^. F7 and EtOAc fractions reduced AGE-specific fluorescence in catalase incubated with fructose. The results of our study showed that the proanthocyanidins-enriched fraction protected the enzyme to some extent against AGEs formation induced by fructose and can, at least in part, negate the effects of glycation on catalase and act as anti-glycation agent.

The *in silico* predicted pharmacokinetic properties showed favorable intestinal absorption ( > 40%) for proanthocyanidins, however, the higher the oligomer the lower its absorption^52^. According to Spencer, *et al*.^53^, the higher the polymerization index of the monomer, the more readily the components are cleaved. Thus, the monomeric and dimeric forms may be the forms presented at highest concentration to the small and large intestine. Proanthocyanidin monomers and dimers, as well as their methylated and glucuronidated forms are the main metabolites of proanthocyanidins in plasma^52^. The results of steady state volume of distribution (VDss) indicate that all proanthocyanidins of F7 can be distributed evenly providing an equal level of blood plasma^54^. Also, the dimer and trimer proanthocyanidins showed the highest values of total clearance, indicating that they are excreted more rapidly than the higher oligomers^54^. Furthermore, there was no negative interference in metabolism, since no inhibition of cytochrome P enzymes such as CYP1A2, CYP2C19, CYP2C9, CYP2D6 and CYP3A4 was detected. With regard to toxicity, all compounds showed no hepatotoxicity or mutagenicity, corroborating data from Yamakoshi, *et al*.^55^, Bak, *et al*.^56^ and Yu and Swaminathan^57^.

In summary, the present study describes the characterization of proanthocyanidins from *A. crassiflora* fruit peel as anti-glycating agent and their mechanism of action. F7 showed high antioxidant and anti-glycation capacities, with inhibitory activities against lipid peroxidation, and AGEs and ROS formation. In addition, there were reductions in AGEs-induced protein carbonyls and protective effects against oxidation of thiol groups and glycated-catalase. The proanthocyanidins were predicted to have good pharmacokinetics properties and no toxicity. The fruit peel of *A. crassiflora*, a species from the Brazilian Savanna, a global biodiversity hotspot, could be potential source of nutraceuticals that offer combined antioxidant and anti-glycation properties to combat the oxidative stress and non-enzymatic glycation, which are present in many pathologies including diabetes *mellitus*.

## Methods

### Drugs and reagents

Reagents and solvents of analytical grade were purchased from Sigma (Sigma, St Louis, MO, USA) or from local suppliers. Water used was Milli-Q obtained by deionized water that has filtered on a millipore filter.

### Preparation of plant material and proanthocyanidins fractions

Fruits of *A. crassiflora* were collected in the north region of Minas Gerais State in March 2017. The plant was identified by André Vito Scatigna from the Biology Institute of the Federal University of Uberlandia, Uberlândia-MG, Brazil. A voucher specimen (HUFU68467) was deposited in the Herbarium Uberlandense. The peels of *A. crassiflora* fruit were separated from pulps, and the dried and powdered peels (7.0 kg) were extracted by the maceration method for three days with 98% ethanol (18 L) in room temperature (25 °C). This process was performed 6 times. After filtration, ethanol was removed using a rotary evaporator (Bunchi Rotavapor R-210, Switzerland) in a water bath at 40 °C under reduced pressure (344.7 g, 4.92%). The crude EtOH extract (100.0 g) was resuspended in MeOH:H_2_O solution (9:1, v v^−1^, 500 mL), filtered (insoluble residue 15.7 g), and submitted to a liquid-liquid extraction according to a protocol designed by Justino, *et al*.^15^ using *n*-hexane (5 × 200 mL, 4.7 g), CH_2_Cl_2_ (5 × 200 mL, 8.3 g), EtOAc (5 × 200 mL, 4.7 g), and *n*-BuOH (5 × 200 mL, 37.0 g). Additionally, an aqueous fraction (15.2 g) was obtained. The extractive solvents were removed using a rotary evaporator under reduced pressure at 40 °C and the fractions were frozen and lyophilized (L101, Liobras, SP, Brazil) to remove the remaining water. EtOAc fraction (2.8 g) was dissolved in MeOH:H_2_O (3.5:6.5) and applied to a size exclusion column (Sephadex LH-20, 500 × 22 mm, GE Health-care Bio-Sciences AB, Uppsala, Sweden) pre-equilibrated with MeOH:H_2_O (3.5:6.5) for 4 h. Initially, sugars and low-molecular-weight phenolic compounds (as flavonols) were eluted from the chromatographic column with MeOH:H_2_O (3.5:6.5, v v^−1^, 500 mL). Further, the proanthocyanidins were eluted with acetone:H_2_O (60:40, v v^−1^; 250 mL)^58^ and the fractions were analyzed by TLC and grouped according to the R_f_ values (12 fractions). The solvents were removed using rotary evaporated under reduced pressure at 40 °C and lyophilized to dryness.

### Phytochemical prospection

#### Total phenolic content

In a 96-well microplate was inserted into each well 5 μL of fractions (500 µg mL^−1^, methanol), 25 μL of Folin-Ciocalteu (10%, water) and 195 μL of water. After 6 min of incubation at 25 °C, 75 μL of Na_2_CO_3_ (7%, water) was added and the mixture was incubated at 25 °C in the dark for 2 h. The absorbance was read at 760 nm (Molecular Devices, Menlo Park, CA, USA)^59^. The total phenolic content was obtained using an analytical curve constructed with gallic acid standards. The results were expressed as milligrams of gallic acid equivalents per gram of sample (mg GAE g^−1^).

#### Total proanthocyanidins content

In a 96-well microplate was inserted into each well 10 µL of each fraction (500 µg mL^−1^, methanol) was incubated with 200 µL of vanilin (4%, methanol) and 100 µL of HCl at 25 °C for 15 min. After incubation, the absorbance was read at 500 nm (Molecular Devices, Menlo Park, CA, USA)^59^. The total proanthocyanidins content was determined using an analytical curve constructed with catechin standards. The results were expressed as milligrams of catechin equivalents per gram of sample (mg CE g^−1^).

#### Liquid chromatography-mass spectrometry analysis

The most active procyanidins-enriched fraction in the biological assays were analyzed by high-performance liquid chromatography-electrospray ionization tandem mass spectrometry (HPLC–ESI–MS) to identify the chemical compounds, according to Justino, *et al*.^60^ and Justino, *et al*.^15^. HPLC Agilent Infinity model 1260 was coupled in Mass Spectrometer Agilent model 6520 B Quadrupole Time of Flight Q-TOF with ESI source. The chromatographic parameters were: 50 × 2.1 mm column, pore diameter of 110 Å and particles of 1.8 µm (Agilent Zorbax model) and a mobile phase containing water acidified with formic acid at 0.1% (v v^−1^) (A) and methanol (B). The gradient solvent system (B) was: 2% (0 min); 98% (0–15 min); 100% (15–17 min); 2% (17–18 min) and 2% (18–22 min), with a flow of 0.35 mL min^−1^ and detection at 280 and 360 nm. The ionization parameters were: 8 L min^−1^ secant gas at 220 °C, nebulizer pressure at 58 psi and 4.5 kVa energy in the capillary. Sequential mass spectrometry (MS/MS) analyzes were performed on different collision energies (5–30 eV) for the positive and negative modes for each molecular ion. The identified compounds were proposed according to the high-resolution spectra of the molecular ions, error values (ppm), retention times, sequential mass spectra and comparisons with data from the literature and Metlin library.

### *In vitro* antioxidant capacity

#### DPPH antiradical activity test

The method using the stable free radical 2,2-diphenyl-1-picrylhydrazyl (DPPH) test was performed according to Justino, *et al*.^60^. Briefly, the fractions (diluted in methanol) were incubated with DPPH (0.06 mmol L^−1^, methanol) at 30 °C for 20 min. Reduction in the absorbance of the mixture was measured at 517 nm (Molecular Devices, Menlo Park, CA, USA). Quercetin was used as a positive control. Scavenging of DPPH radicals was calculated using the following formula: Antioxidant activity (%) = [(Abs DPPH – Abs sample)/Abs DPPH] x 100, where Abs DPPH is the absorbance of DPPH radical and Abs sample is the absorbance of the DPPH radical in presence of fraction/control. A blank was performed with the fraction/control to reduce the contribution of the color to the absorbance.

#### ORAC antioxidant activity test

The method of oxygen radical absorbance capacity (ORAC) was performed according to Justino, *et al*.^60^. The fractions (diluted in methanol) were incubated with fluorescein (0.085 nmol L^−1^, phosphate buffer, 75 mmol L^−1^, pH 7.4) at 25 °C for 15 min. 2,2′-Azobis-(2-amidino-propane)-dihydrochloride (153 mmol L^−1^, phosphate buffer, 75 mmol L^−1^, pH 7.4) was added to the mixture and the loss of fluorescence intensity was measured at 37 °C for 90 min, by calculating the area under the curve (485 nm_ex_/528 nm_em_) (Perkin-Elmer LS 55, Massachusetts, USA). The antioxidant capacity was determined using an analytical curve constructed with trolox as standard (µmol TE/g). Quercetin was used as a positive control.

#### FRAP antioxidant activity test

The method of ferric reducing antioxidant power (FRAP) was performed according to Benzie and Strain^61^. Briefly, the fractions (diluted in methanol) were incubated at 37 °C for 6 min with FRAP reagent containing the following reagents in 10:1:1 proportion: sodium acetate buffer (300 mmol L^−1^, pH 3.6), 2,4,6-tris(2-pyridyl)-s-triazine (10 mmol L^−1^) and ferric chloride (20 mmol L^−1^). The absorbance was measured at 593 nm (Molecular Devices, Menlo Park, CA, USA) and the antioxidant activity was determined using an analytical curve constructed with trolox as standard (µmol TE g^−1^). Quercetin was used as a positive control. A blank was performed with the fractions/control to reduce the contribution of the color to the absorbance.

#### Generation of bone marrow-derived macrophages (BMDM)

This method was based on Larmonier, *et al*.^62^. Macrophages were obtained by differentiation of bone marrow cells from 6–8 week old male C57BL/6 mice. Bone marrow cells were extracted by flushing bone cavity with RPMI 1640 into a 15 mL sterile polypropylene tube. The cells were homogenized and cell suspension was centrifuged (400 *xg* for 8 min, 4 °C). The pellet was homogenized in RPMI-1640 supplemented with 20% of fetal bovine serum (Gibco), 2 mmol L^−1^ L-glutamine, 100 U mL^−1^ penicillin, 100 µg mL^−1^ streptomycin and 30% L929-cell conditioned medium (LCCM), which was used as a source of macrophage colony stimulating factor (M-CSF). Cells were kept at 37 °C and 5% CO_2_ atmosphere; 5.0 × 10^6^ cells were cultured to generate BMDM. BMDM were collected after 4 days by removing the supernatant of culture dishes and detaching adherent cells using sterile ice-cold phosphate-saline buffer (PBS). The animal proceedings were all previously approved by institutional Animal Ethics Committee (protocol number 032/18).

#### Cell viability

This method was based on Lee and Park^63^. BMDM were suspended in RPMI-1640 supplemented in according with aforementioned, except LCCM which was not added. Cells were seeded in 96-well microplate at density of 0.2 × 10^6^ cells/well and were treated for 24 h with the fractions (diluted in RPMI-1640). Next, the supernatant was removed and incubated with 100 µL of 5 mg mL^−1^ MTT (3-(4,5-dimethylthiazolyl-2)−2,5-diphenyltetrazolium bromide) solution at room temperature for 2 h. The purple formazan formed by viable cells were solubilized in dimethyl sulfoxide (DMSO) and the absorbance was measured at 570 nm (Molecular Devices, Menlo Park, CA, USA).

#### Inhibition of ROS production

This method was based on Shih, *et al*.^64^. Briefly, BMDM cells, previously diluted in Hank’s Balanced Salt Solution (HBSS) without phenol red (Sigma-Aldrich), were seeded to white opaque 96-well microplate at a density of 0.2 × 10^6^ cells/well. Cells were pre-incubated with the fractions (diluted in HBSS) for 30 min at 37 °C and 5% CO_2_ atmosphere. Next, luminol (final concentration of 625 nmol L^−1^) was added to each well and the generation of ROS was induced by opsonized- zymosan from *Saccharomyces cerevisiae* (final concentration of 100 µg mL^−1^). Opsonized-zymosan was previously prepared by incubating zymosan with serum of mice for 30 min at 37 °C. Zymosan suspension was centrifuged at 400 *xg* for 8 min and the pellet was homogenized in HBSS. The generation of ROS by BMDM was monitored for 4 h through chemiluminescence emission resulting from luminol oxidation by using a microplate reader (Perkin-Elmer LS 55, Massachusetts, USA). Results were expressed as Relative Luminescence Units (RLU).

#### Inhibition of non-enzymatic lipid peroxidation

The inhibition of Fe^2+^-ascorbate-induced lipid peroxidation was assayed by a modified method of Justino, *et al*.^60^. Male Wistar rats (200–270 g) were housed in a controlled environment (22 ± 2 °C) with a 12-h light/dark cycle (lights on between 6:00 AM and 6:00 PM) and fed standard laboratory chow and tap water *ad libitum*. All animal experiments were complied with the ARRIVE guidelines and approved by the Ethics Committee in Animal Experimentation of the Federal University of Uberlândia (CEUA/UFU-015/17). The liver tissues were removed and homogenized in phosphate buffer (1:10 w v^−1^, pH 7.4), then centrifuged at 3000 × *g* for 10 min at 4 °C. Lipid peroxidation was determined according to the thiobarbituric acid reactive substances (TBARS) method. Liver homogenate (0.5 mL) was incubated with phosphate buffer (0.9 mL), 0.1 mmol L^−1^ FeSO_4_.6H_2_O (0.25 mL), 0.1 mmol L^−1^ ascorbic acid (0.25 mL) and fractions/control (0.1 mL, diluted in water) at 37 °C for 60 min. Then, the mixture was incubated with 1 mL of 10% TCA (w v^−1^) and 1 mL of 0.67% thiobarbituric acid (in 7.1% Na_2_SO_4_) at 100 °C for 15 min. The samples were centrifuged at 3000 × *g* for 15 min and the absorbance of the complex was measured at 532 nm (Molecular Devices, Menlo Park, CA, USA). The supernatant was also used for FRAP assay (see methodology above). Quercetin was used as a positive control.

### Inhibition tests for the formation of advanced glycation end products (AGEs)

#### BSA-fructose assay

The anti-glycation assay using the BSA-fructose model was performed according to Franco, *et al*.^65^. The fractions (diluted in methanol) were incubated in the dark with 50 mg mL^−1^ BSA, 1.25 mol L^−1^ fructose and 200 mmol L^−1^ sodium phosphate buffer containing 0.02% sodium azide, pH 7.4, at 37 °C for 72 h. Control samples without fructose and/or fractions were incubated under the same conditions. After incubation, 20% TCA (w v^−1^) was added and the mixture was centrifuged at 10,000 × *g* for 10 min. The pellet was resuspended in phosphate buffer and the fluorescence intensity was measured at 350 nm_ex_/420 nm_em_ (Perkin-Elmer LS 55, Massachusetts, USA). Quercetin was used as a positive control and methanol as a negative control. The blank was carried substituting fructose by phosphate buffer. The results were presented as a percentage of glycation inhibition (GI), calculated according to the following equation:

$${\rm{GI}}\,( \% )=100-[({\rm{F}}\,{\rm{sample}}-{\rm{F}}\,{\rm{blank}})/({\rm{F}}\,{\rm{control}}-{\rm{F}}\,{\rm{blank}})]\times 100,$$

where: F sample is fluorescence intensity in presence of fraction/control; F blank is fluorescence intensity in the absence of fructose and fraction/control; and F control is fluorescence intensity in the absence of fraction/control.

#### BSA-methylglyoxal assay

The anti-glycation assay using the BSA-methylglyoxal model was performed according to Justino, *et al*.^60^. The fractions (diluted in methanol) were incubated in the dark with 50 mg mL^−1^ BSA, 53.3 mmol L^−1^ methylglyoxal and 200 mmol L^−1^ sodium phosphate buffer containing 0.02% sodium azide, at 37 °C for 72 h. Control samples without fructose and/or fractions were incubated under the same conditions. After incubation, 20% TCA (w v^−1^) was added and the mixture was centrifuged at 10000 × *g* for 10 min. The pellet was resuspended in phosphate buffer and the fluorescence intensity was measured at 340 nm_ex_/380 nm_em_ (Perkin-Elmer LS 55, Massachusetts, USA)^30^. Quercetin was used as a positive control and methanol as a negative control. The blank was carried substituting methylglyoxal by phosphate buffer. The results were presented as a percentage of glycation inhibition, using the same equation in BSA-fructose model.

#### Arginine-methylglyoxal assay

The anti-glycation assay using the arginine-methylglyoxal model was performed according to Justino, *et al*.^60^. The fractions (diluted in methanol) were incubated in the dark with 106.6 mmol L^−1^ arginine, 53.3 mmol L^−1^ methylglyoxal and 200 mmol L^−1^ sodium phosphate buffer containing 0.02% sodium azide, at 37 °C for 72 h. Control samples without fructose and/or fractions were incubated under the same conditions. After incubation, the fluorescence intensity was measured at 340 nm_ex_/380 nm_em_ (Perkin-Elmer LS 55, Massachusetts, USA). Quercetin was used as a positive control and methanol as a negative control. The blank was carried substituting methylglyoxal by phosphate buffer. The results were presented as a percentage of glycation inhibition, using the same equation in BSA-fructose model.

#### Measurement of protein-bound carbonyl and thiol groups of glycated BSA

BSA at 50 mg mL^−1^ was incubated in the dark with 1.25 mol L^−1^ fructose with or without the fractions (diluted in methanol) in 200 mmol L^−1^ sodium phosphate buffer containing 0.02% sodium azide, pH 7.4, at 37 °C for 4 days. Control sample without fructose and fractions was incubated under the same conditions. Carbonyls in BSA and in glycated BSA in the presence or absence of fractions were identified by 2,4-dinitrophenylhydrazine (DNPH), precipitated with 20% TCA, washed with ethanol-ethyl acetate and dissolved in 6 mol L-1 guanidine hydrochloride^66^. The absorbance values were registered at 370 nm (Molecular Devices, Menlo Park, CA, USA) and the carbonyl content was calculated using a molar absorbance of 22,000 mol L^−1^ cm^−1^; results were expressed as the ratio of nmols of DNPH reacted. Free thiol content was determined in BSA and in glycated BSA in the presence or absence of fractions according to an established method using 5,5′-dithiobisnitrobenzoic acid (DTNB)^67^. Free thiol concentration was calculated using 14,150 mol L^−1^ cm^−1^ as the molar extinction coefficient of DTNB.

#### Crosslinked advanced glycation end-products (AGEs)

BSA or lysozyme (10 mg mL^−1^) were incubated in the dark with 0.5 mol L^−1^ fructose or 53.3 mmol L^−1^ methylglyoxal with or without the fractions in 200 mmol L^−1^ sodium phosphate buffer containing 0.02% sodium azide, pH 7.4, at 37 °C for 30 days. Control samples without fructose and/or fractions were incubated under the same conditions. Crosslinked AGEs were assessed by crosslinking of the protein following sodium dodecyl sulphate-polyacrylamide gel electrophoresis (SDS-PAGE) using 10% gels as described previously^37^. These gels were stained with Coomassie blue, destained and photographed (Amersham Imager 600 system, GE Healthcare Bio-Sciences AB, Uppsala, Sweden).

#### Measurement of glycated catalase activity and fluorescence

Measurement of glycated catalase activity was performed according to Mofidi Najjar, *et al*.^49^, with modifications. Bovine liver catalase (0.175 mg mL^−1^) was incubated with 16 mmol L^−1^ fructose and with or without the fractions in 10 mmol L^−1^ potassium phosphate buffer, pH 7.0, at 37 °C in the dark for 96 h. Control samples without fructose and/or fractions was incubated under the same conditions. Catalase activity evaluation was based upon hydrogen peroxide (0.2%) decomposition^68^ at 240 nm for 5 min (Perkin-Elmer LS 55, Massachusetts, USA). In addition, the fluorescence intensity of catalase and glycated catalase in the presence and absence of the fractions was measured at 350 nm_ex_/420 nm_em_ 96 h after incubation (Perkin-Elmer LS 55, Massachusetts, USA).

#### *In Silico* pharmacokinetic properties prediction

The pharmacokinetic properties of the procyanidins identified in *A. crassiflora* fruit peel were predicted by pKCSM^54^. These signatures were used to evaluate intestinal absorption, steady state volume of distribution (Vdss), cytochrome p inhibition, total clearance, mutagenicity and hepatotoxicity.

#### Statistical analysis

The statistical analyzes and graphics were done using GraphPad Prism 6.0 software. All analyzes were performed in triplicate and the data were expressed as mean ± standard deviation. The significance of difference was calculated using one-way and two-way ANOVA, and Dunnett’s and Tukey’s post-tests for multiple comparisons. Pearson’s correlation was used to examine linear relationships between the results. Values of *p* < 0.05 were considered significant.

## Supplementary information

Supplementary information

## Data Availability

All data generated or analyzed during the current study are included.
