# Supplementary material for: B procyanidins of Annona crassiflora fruit peel inhibited glycation, lipid peroxidation and protein-bound carbonyls, with protective effects on glycated catalase
Source: Sci Rep. 2019 Dec 16;9:19183. doi: 10.1038/s41598-019-55779-3 (PMC6915705; doi:10.1038/s41598-019-55779-3)
Supplement: Supplementary file 1 — Supplementary information [file 41598_2019_55779_MOESM1_ESM.docx]

**B procyanidins of *Annona crassiflora* fruit peel inhibited non-enzymatic glycation, lipid peroxidation and protein-bound carbonyls, with protective effects on glycated catalase**

Allisson B Justino^1^, Rodrigo R Franco^1^, Heitor C G Silva^1^, André L Saraiva^1^, Raquel M F Sousa^2^, Foued S Espindola^1,*^

^1^Institute of Biotechnology, Federal University of Uberlandia, Av. Pará, 1720, 38400-902, Uberlândia/MG – Brazil.

^2^Institute of Chemistry, Federal University of Uberlandia, Av. João Naves de Ávila, 2121, 38408-100, Uberlândia/MG – Brazil.

*Corresponding author:

Foued Salmen Espindola

E-mail: foued@ufu.br

Phone: 55 (34) 3225-8439

Address: Universidade Federal de Uberlândia, Instituto de Biotecnologia, Av. Pará, 1720, CEP 38400-902, Uberlândia-MG, Brasil.

**Appendices**

 **Fig. S.1.** Chromatogram of the proanthocyanidins-enriched fraction from *A. crassiflora* fruit peel by HPLC-ESI-MS/MS (negative mode).

**Fig. S.2.** HPLC-ESI-MS/MS of B-type procyanidin (dimer) from the proanthocyanidins-enriched fraction (*m*/*z* 577 [M-H]^-^).

**Fig. S.3.** HPLC-ESI-MS/MS of B-type procyanidin (trimer) from the proanthocyanidins-enriched fraction (*m*/*z* 865 [M-H]^-^).

 **Fig. S.4.** HPLC-ESI-MS/MS of B-type procyanidin (tetramer) from the proanthocyanidins-enriched fraction (*m*/*z* 1153 [M-H]^-^).

**Fig. S.5.** HPLC-ESI-MS/MS of B-type procyanidin (tetramer) from the proanthocyanidins-enriched fraction (*m*/*z* 576 [M-2H]^2-^).

**Fig. S.6.** HPLC-ESI-MS/MS of B-type procyanidin (pentamer) from the proanthocyanidins-enriched fraction (*m*/*z* 720 [M-2H]^2-^).


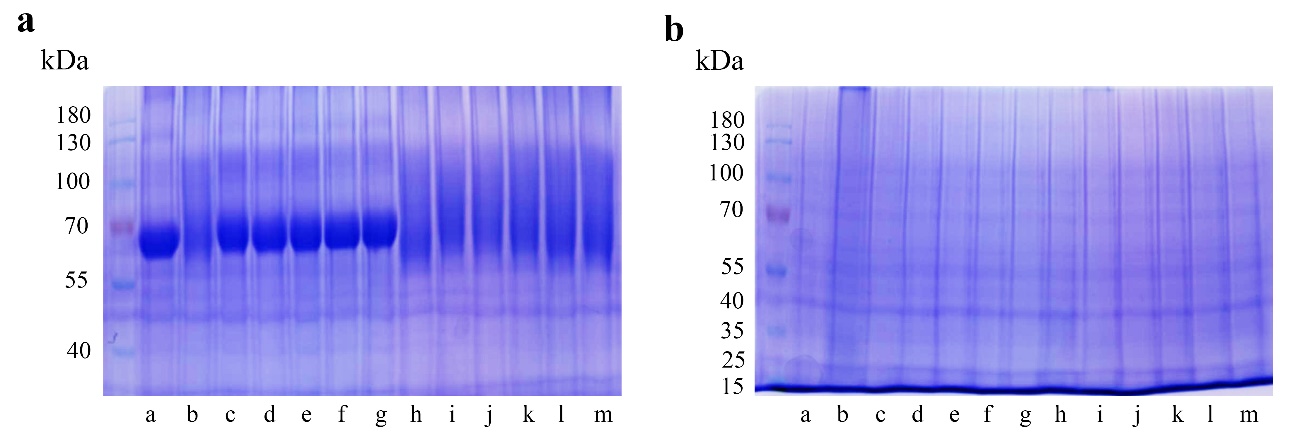


**Fig. S.7.** SDS-PAGE gels showing the effects of quercetin on formation of crosslinked AGEs formed by glycation of BSA (10 mg mL^-1^) (a) and lysozyme (10 mg mL^-1^) (b) by fructose (lanes b-g) or methylglyoxal (lanes h-m) for 4 weeks at 37 °C. Unmodified BSA and lysozyme (lane a); glycated BSA and lysozyme by fructose (lane b) or methylglyoxal (lane h); BSA and lysozyme glycated by fructose in the presence of 625 (lane c), 345 (lane d), 187.5 (lane e), 62.5 (lane f) and 34.5 µg mL^-1^ (lane g) quercetin; and BSA and lysozyme glycated by methylglyoxal in the presence of 625 (lane i), 345 (lane j), 187.5 (lane k), 62.5 (lane l) and 34.5 µg mL^-1^ (lane m) of quercetin.
